# Thickness of a metallic film, in addition to its roughness, plays a significant role in SERS activity

**DOI:** 10.1038/srep11644

**Published:** 2015-06-29

**Authors:** Changwon Lee, Christopher S. Robertson, An H. Nguyen, Mehmet Kahraman, Sebastian Wachsmann-Hogiu

**Affiliations:** 1 Center for Biophotonics, University of California, Davis, Sacramento, CA 95817, USA; 2 Department of Chemistry, Faculty of Arts and Sciences, University of Gaziantep, Gaziantep 27310, Turkey; 3 Department of Pathology and Laboratory Medicine, University of California, Davis, Sacramento, CA 95817, USA

## Abstract

In this paper we evaluate the effect of roughness and thickness of silver film substrates, fabricated on glass and polydimethylsiloxane (PDMS) templates, on surface-enhanced Raman Spectroscopy (SERS) activity. While the silver substrates obtained on glass templates exhibit nm-scale roughness, the silver substrates on PDMS templates show larger roughness, on the order of 10 s of nm. These roughness values do not change significantly with the thickness of the silver film. The SERS intensities of 4-aminothiophenol (ATP) deposited on these substrates strongly depend on both roughness and thickness, with more significant contribution from the roughness on thinner films. FEM simulations of the electric field intensities on surfaces of different thicknesses for rough and flat surfaces suggest higher localized plamons on thinner, rough surfaces. This study indicates that, besides roughness, the thickness of the metallic layer plays a significant role in the SERS activity.

Ever since the discovery of the SERS (Surface-Enhanced Raman Spectroscopy) in 1974^1^, its unique characteristics, such as the ability to provide molecule-specific fingerprint-like spectra and the non-destructive, label-free, and highly sensitive nature of measurement, have driven a large number of studies focused on both development of novel plasmonic structures as well as applications of SERS to problems related to molecular detection and characterization^2^,^3^,^4^,^5^. Since the late 1990 s, SERS has particularly benefited from advances in the nanotechnology field, which enabled the development of highly sensitive SERS substrates^6^,^7^,^8^,^9^. Currently, significant efforts by the scientific community are dedicated towards applications of SERS to biomedical problems, food industry, material characterization, environment monitoring, or detection of bio-and chemical hazards. Specifically, to highlight just a few out of many applications^10^,^11^, it has been demonstrated to be useful for the detection of residual pesticides in food^12^, chemical compounds such as melamine and folic acid^7^, measurements of proteins and protein complexes^13^,^14^, cellular characterization^15^,^16^,^17^. On the other hand, the development of SERS substrates is mainly concentrated on novel metallic nanostructures that can be either colloidal particles or nanostructures of various shapes and sizes fabricated on 2D or 3D surfaces^18^. While any noble metal can produce significant SERS enhancement, silver and gold are predominantly used for the fabrication of SERS substrates as silver exhibits the largest enhancement in the visible range and gold is biologically inert^19^.

Despite the fact that colloidal silver substrates exhibit some of the highest reported SERS enhancement factors, the intensity fluctuation of the SERS signal due to Brownian motion and diffusion of clusters through the scattering volume during the measurement (in case of solution phase analysis) and irregular “hot-spot” location of aggregated colloids on the dried surface (in case of solid phase analysis), it is difficult to perform quantitative measurements using such substrates. In addition, colloidal SERS substrates made of silver are stabilized with a reducing agent such as sodium citrate^20^,^21^,^22^,^23^, which adds an electric charge (usually negative) on the surface of the nanoparticles. This same-sign charge keeps the particles separated and attracts molecules of opposite charge. Meanwhile, it repulses the molecules carrying the same kind of charge as the charge on the surface and creates a fundamental problem for quantitative measurements of such molecules. These problems can be minimized by using 2D and 3D SERS structures fabricated on planar substrates via lithographic processes. In this case, a thin metallic layer is deposited via sputtering or other deposition methods on nanostructured templates. Here we understand 2D structures as those created from nanometer-sized structures that are smaller than the wavelength of light, and 3D structures as those that are generated from nano-to micro-meter structures larger than the wavelength of light. Many of lithographic methods, such as electron beam lithography or ion beam lithography are successful in preparing complex 2D and 3D substrate shapes including pillar^24^ and donut-shaped structures^25^ with high SERS enhancement. However, these methods are often time consuming and expensive, and not yet suitable for mass production, which limits their application. In an effort to address this issue, significant research is dedicated towards the preparation of highly SERS active, scalable planar substrates. Recently, we developed planar 3D metallic nanovoid SERS substrates using nanosphere soft lithography that show high geometrical uniformity and reproducible SERS enhancement factors with less than 20% variations in the SERS signal strength over different regions of the substrate^26^. Since the sputtered silver surface does not require any stabilizing ligand, the resulting substrate is charge-free and can be used for the detection of molecules regardless of their charge.

Among the main factors contributing to the SERS enhancement factor of 2D and 3D substrates, surface roughness, and the type of metal have been consistently studied in the literature from late 1970 s which shows that there are rather complex relations between the surface roughness and SERS intensity^27^,^28^,^29^,^30^. For example, Zhao *et al*. showed the effect of roughness on 100 nm-thick silver film on SERS as they observed higher activity for rougher surfaces due to the generation of more hot-spots^29^. In addition, the effect of surface roughness on plamonic gold nanoparticles was investigated by Trugler *et al*. This study indicated that the roughness features on nanoparticles surface could dominate the overall SERS activity^30^. And recently while this article was under-review, the article on the effect of silver thickness on SERS activity focused on hot spots generated at the edge of the sharp 3-D strucutre has been published^31^. However, there are no studies so far aimed at understanding the thickness of the continuous film itself and the interplay between the film thickness and the roughness of the substrate on the SERS enhancement.

In this paper we investigate the hypothesis that both surface roughness and metal thickness of 2D and 3D substrates have a strong effect on the SERS activity. Experimental results are compared with computer simulations to provide a detailed explanation of the observed phenomena. Understanding the role of these parameters in SERS will provide new opportunities not only for the manufacturing of simple, robust, and cost effective substrates, but also for wider applications of these substrates.

## Results And Discussion

**Figure 1:**
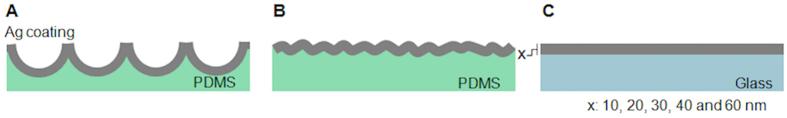
Schematic diagram of the silver nanofilms sputtered on different templates (**A**) 3D silver nanovoid substrate on PDMS template, (**B**) 2D silver flat substrate on PDMS template and (**C**) 2D silver flat substrate on glass slide.

SERS activity of the 2D and 3D substrates prepared for this study is determined by several parameters: (i) nm-sized clusters formed during the sputtering process, (ii) 10 s of nm sized rough structures formed on PDMS during the vacuum phase of the sputtering process, (iii) the thickness of the thin film, and for the 3D nanovoid structures, (iv) the nanovoid structure formed by the polystyrene latex beads in the PDMS template. To investigate the specific role of, as well as the interplay between these parameters, we prepared three different planar silver sputtered substrates, (A) 3D nanovoid substrate on PDMS template [to address parameters (i), (ii), (iii) and (iv)], (B) 2D substrate on PDMS template [for parameters (i), (ii) and (iii)] and (C) 2D substrate on glass slide [for parameters (i) and (iii)]. A schematic description of these substrates is presented in Fig. 1. To distinguish nanovoid areas from non-nanovoid areas of PDMS templates, we used the term “flat PDMS“ to indicate non-nanovoid areas. Please note that it does not indicate absolute flat surface.

**Figure 2:**
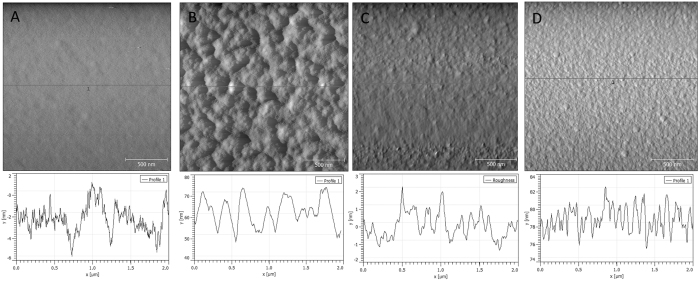
AFM images and height profiles of different surfaces (**A**) flat PDMS template without the sputtering process (2 μm x 2 μm), (**B**) 20 nm thick 2D silver substrate on flat PDMS template (2 μm x 2 μm), (**C**) glass slide surface before silver sputtering and (**D**) 20 nm thick 2D silver substrate on glass slide (2 μm x 2 μm).

Three-dimensional surface roughness of the silver substrates and templates are analyzed by AFM. The image of flat PDMS before the sputtering process shows a relatively flat surface (Fig. 2A) while the silver sputtered flat PDMS surface shows a rougher surface with bumps and wrinkles (Fig. 2B). The glass slide surface before the sputtering process showed mild roughness (Fig. 2C) and silver sputtered surface on glass slide showed nm range globular domains (Fig. 2D). These data suggest that the sputtering process generates rough surfaces composed of globular clusters of a few nm ranges. In fact, similarly prepared silver nanofilms by vacuum evaporation also showed a few nm range clusters which showed weak SERS activity by itself even when sputtered on flat surfaces^30^. This type of surface roughness is well depicted in the AFM pictures of the sputtered silver nanofilm on glass slide shown in Fig. 2D and Figure S1. The surface roughness of the silver film on flat glass slide showed a few nm, mostly less than 5 nm, regardless the thickness of the sputtering as shown in Figure S1A~C. Meanwhile, on top of the roughness created by sputtering process, flat PDMS surface showed additional roughness of dense wrinkles and bumps throughout the surface which is deformed during the high-vacuum phase of the sputtering process (Figure S1D~F). The wrinkles created during the high-vacuum condition are up to 100 nm in depth and several μm in length, while the bumps have widths of 200~300 nm with heights of around 20 nm. These are shown in the large area AFM scan (20 × 10 μm) of Figure S2. AFM images of flat PDMS template without the sputtering process show smooth flat surfaces without any additional roughness formation (Fig. 2A). However, even on this smooth surface of flat PDMS, the height profile shows numerous little bumps in the range of 1 ~ 2 nm. These little bumps are believed to be formed by surface mobility of glassy polymers which could happen well below the glass transition temperature (T_g_) as described by Chai *et al*.^31^ During the sputtering process, additional irregularities are added as globular clusters (from the metallic film) and wrinkles and bumps (from the deformation of the PDMS template under vacuum conditions). These surface irregularities were analyzed by AFM and are shown in Fig. 2B, S1D–S1F and S2.

To further investigate the effect of the sputtering process on the surface roughness and ultimately on the SERS activity, three different silver sputtered planar substrates, on nanovoid PDMS template, flat PDMS template and flat glass slide, were prepared. A typical SEM image of 3D silver nanovoid substrate on PDMS template is shown in Figure S3. As it is shown in Fig. 2D, the silver sputtered on flat glass presents a wrinkle-free surface composed of few nm sized globular domains. Meanwhile, the silver film deposited on a flat PDMS template surface shows larger sized wrinkles and bumps. As the AFM image of PDMS flat surface without sputtering process is very smooth and flat without any wrinkles (just like the glass slide), this further indicates that the additional surface roughness of the silver sputtered surface was created during the sputtering process. The sputtering process requires high vacuum condition (10 mTorr) which can cause deformation of the previously flat PDMS surface. This deformation could possibly be due to degassing of entrapped air in the PDMS polymer network and hardening via surface coating and additional polymerization of unreacted monomer in the polymer network by the high UV exposure in the sputtering chamber.

To quantitatively analyze the surface roughness we used Gwyddion software. The arithmetic average of absolute values, *R*_*a*_, was calculated using 3-dimensional roughness data obtained from AFM analysis for the 2D silver flat substrate on PDMS template and glass slide with thicknesses of 10, 20 and 40 nm, and the results are shown in Table 1. *R*_*a*_ is defined by the following equation, 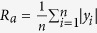
31. The values presented in Table 1 indicate that silver nanofilms sputtered on flat PDMS template has 5 to 10 times higher R_a_ values comparing to silver nanofilms sputtered on glass slide. The R_a_ value of untreated flat PDMS is, on the other hand, similar to silver nanofilms on glass slide. The smoothest surface is the glass slide with an R_a_ value of 0.57 nm. In conclusion, the surface roughness of both glass slide and PDMS template does not change significantly with the thickness of the silver film. The R_a_ value of the nanovoid structure of 89.6 nm is an order of magnitude higher than the R_a_ values of flat substrates. This R_a_ value is determined by the micrometer scale curvature of the structure and is not comparable to the R_a_ of the flat substrates which are determined by the roughnees of the surface.

In addition to roughness, another important parameter that we hypothesized to play a significant role in SERS is the thickness of the metallic film. This hypothesis assumes a continuous film layer with thickness as small as 10 nm. If the film is not continuous and forms individual globular clusters as reported in Perumal *et al*., the origin of increased SERS activity is hot-spots generated by individual nanoclusters rather than the thickness and surface roughness effects^32^. In their study, Perumal *et al*. created a thin film of silver using vacuum evaporation method and showed that granular silver clusters have been formed at thickness smaller than 20 nm before it eventually forms continuous layer of silver film at larger thicknesses^32^. However, in our study we use sputtering to create continuous nanofilms that can be controlled with nm accuracy, as demonstrated by AFM measurements. In Figure S4 we describe the process of measuring the continuity and thickness of the silver thin films. Scotch tape is used to cover a portion of glass slide surface prior to the sputtering process and the tape was removed after the sputtering to determine the thickness of the silver deposition. As the tape removed upward, the silver film layer at the edge has been lifted and formed a ridge along the edge area. The AFM image in Figure S4 shows that a 10 nm-thick silver sputtering set up results in a continuous film that is slightly larger, up to approximately 12–13 nm in thickness.

**Figure 3:**
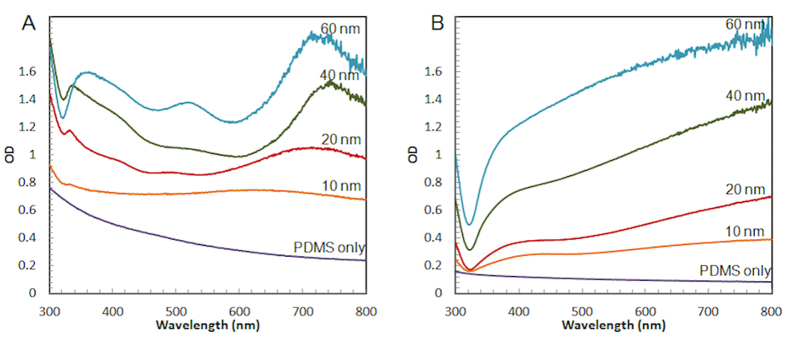
UV-Vis absorption spectra of nanovoid and flat silver substrates, (**A**) 3D nanovoid and (**B**) 2D flat on PDMS template with silver thicknesses of 0, 10, 20, 40 and 60 nm.

In order to identify the plasmon resonances in the 2D and 3D structures, we performed absorption spectroscopy on substrates placed perpendicular on the light beam inside a Varian Cary spectrometer. Fig. 3A shows the absorption spectra of 3D nanovoid structures for different silver thicknesses, and Fig. 3B shows absorption spectra of 2D flat PDMS with silver deposited at different thicknesses. There are two main absorption bands observed in typical 3D silver nanovoid substrates as we have shown in our previous study^26^. The band at the shorter wavelengths (around 500 nm) is called °D mode and originates from the surface plasmons confined near the bottom of the void. The °D mode at 500 nm is only clearly visible at 60 nm layer silver nanovoid. The absorption band located at lower wavelengths (around 750 nm) is called °P mode and is due to the plasmons generated near to the top center of the void. In Fig. 3A we show that the nanovoids exhibit clear °D and °P mode absorption peaks at 40 and 60 nm thick silver nanovoid structures. We note that the °P peak appears at slightly longer wavelengths for the 40 nm silver layer compared with the 60 nm layer, possibly due to slightly larger bowl size resulted by the thinner silver coating. In addition, the 40 nm nanovoid substrate has a less defined °D peak, and as the silver layer gets thinner, both °D and °P peaks become less defined and almost not identifiable at 10 nm thickness. By comparison, the 2D flat silver substrate on PDMS template does not exhibit distinct absorption band and the absorption increases uniformly throughout the visible range when the silver thickness increases (Fig. 3B).

**Figure 4:**
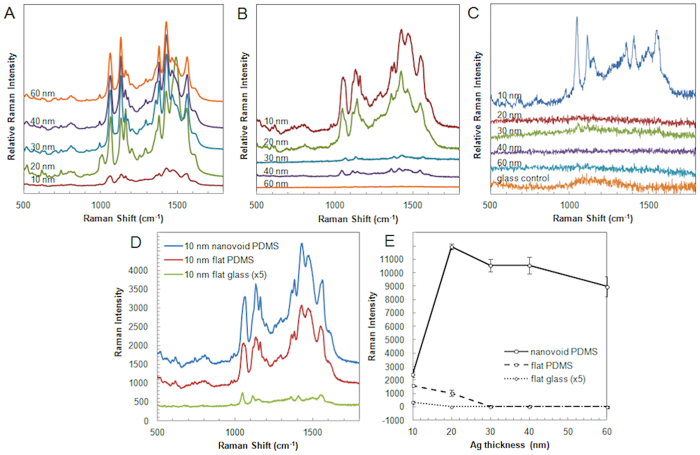
SERS activity measured using 4-ATP as reporter molecule on different silver nanofilm substrates (**A**) SERS spectra measured on 3D silver nanovoid substrates on PDMS template, (**B**) SERS spectra measured on 2D flat silver substrate on PDMS template, (**C**) SERS spectra on 2D flat silver substrate on glass slide (**D**) comparison of SERS spectra on 2D and 3D 10 nm silver substrates and (**E**) SERS intensity dependence on silver thickness with the different silver film thicknesses (10, 20, 30, 40 and 60 nm) measured for the 1050 cm^-1^ peak on 2D and 3D silver substrates. Incident light of 633 nm laser at 42 μW (A, B) and 210 μW (**C**) was used.

In order to characterize the SERS enhancement of substrates with different silver roughness and thickness, we performed SERS measurements using 4-ATP as a reporter molecule. 4-ATP was used as its thiol group has very high affinity to silver to form stable Ag-S bonds, and forms a uniform layer on the surface^33^. The concentrations of the surface adsorbed 4-ATP molecule between the different substrates are the same as the entire substrate was immersed inside the excessive 4-ATP solution for the same amount of time (1.5 hour) to saturate the metallic surface. Several SERS peaks of 4-ATP are obtained, and among them the 1050 cm^-1^ peak (v-CS) was chosen to compare the SERS intensity obtained under different conditions. In Fig. 4 we show the dependence of the SERS intensity of this peak on silver film thickness and roughness for 2D and 3D structures. Nanovoid (3D) structures exhibit the highest SERS signal at the thickness of 20 nm and show a gradual decrease of intensity as the silver layer becomes thicker (Fig. 4A). While the intensity has decreased by 25% at the thickness of 60 nm, it has dropped by 79% at the thickness of 10 nm. Meanwhile, the SERS intensity of rough flat silver surface on the PDMS template showed the highest SERS activity at the thinnest silver layer of 10 nm and showed negligible SERS activity for layers thicker than 30 nm (Fig. 4B). At small thickness we believe that the plasmons are more localized, leading to higher enhancements. In this case, in 2D, 10 nm rough flat silver substrate on PDMS template, SERS activity originates from combined contributions from (i) the rough surface with nanostructures such as a few nm sized globular domains and larger scaled wrinkles and bumps formed during the sputtering process by the deformation of the PDMS template and (ii) the localization of the plasmon on very thin silver coatings.

To isolate and evaluate the effect of the wrinkles and bumps (parameter (ii) formed during the sputtering process of PDMS) on the SERS activity, silver was coated on top of the glass slide, which does not deform nor degas during the sputtering process, and the spectra compared with those obtained on PDMS. As it is shown in Fig. 2D, the silver-coated glass surface appears to be composed of sub-10 nm globular domains without any large wrinkles and bumps. Thus, this 2D flat substrate only has two possible parameters influencing SERS activity, silver thickness and sub-10 nm globular domains. Representative SERS spectra obtained for different silver thicknesses in silver are shown in Fig. 4C. A comparison of these spectra with those obtained on PDMS is shown in Fig. 4D. At 10 nm silver thickness, the SERS intensity of the 2D PDMS structures is approximately 66% of the SERS intensity of the 3D nanovoid structures, suggesting that the effect of 3D nanovoid structure is minimal at 10 nm thickness and it only starts to dominate for layers thicker than 20 nm.

The additional SERS enhancement for 3D nanovoid structures compared with 2D structures with similar roughness could be attributed to Bragg and Mie scattering that may occur in this situation due to the larger scale 3D nanovoid structures^34^. The additional scattering by these 3D structures generates progating and localized plasmons that will contribute to enhanced SERS activity, as observed in our experiments. This hypothesis is further supported by FEM simulations that are presented in the supplementary materials (Figure S7). In addition, the SERS activity from the 2D flat silver substrate on glass slide is weaker compared to the 2D flat silver substrate on PDMS template. In order to obtain reliable SERS spectra, we used 210 μW of the 633 nm laser for the glass slide substrate, which is 5 times stronger than the laser power (42 μW) used for the PDMS-based substrates. In case of the 10 nm thickness, the SERS intensity of the flat silver substrate on glass slide is 35 times weaker than the SERS intensity of the flat silver substrate on PDMS template of the same thickness. In addition, for flat silver substrate on glass slide with thicknesses larger than 20 nm we could not measure any SERS spectra. This result suggests that the sub-10 nm globular domains formed during the sputtering only have very limited effect of SERS process. Hence the major effect contributing the SERS activity of 10 nm flat silver substrate on glass slide is the localized plasmon originated in thin silver film. This further suggests that the effect of the rough surface created on PDMS during the sputtering process is responsible for most of the SERS activity exhibited at 10 nm thickness on both PDMS template based 2D flat silver substrates and 3D silver nanovoid substrates.

To summarize these phenomena in a single graph, we plotted the SERS intensities of the 1050 cm^-1^ peak measured for different silver thicknesses on nanovoid PDMS, flat PDMS, and flat glass templates (Fig. 4E). The SERS intensity of the 10 nm flat silver substrate on glass (R_a _= 1.2 nm) is 35 times weaker than the intensity from the flat silver substrate on PDMS template of the same thickness (R_a_ = 9.3 nm). This observation indicates that nm sized globular domains (parameter (i)), only plays a negligible role on SERS activity and the intensity difference between the two substrates mainly originates from the additional roughness of the silver sputtered flat PDMS substrate (parameter (ii)) when interplayed with the thin layer of silver (10 nm) (parameter (iii)).

The comparison of the SERS activity of 2D flat silver substrates on PDMS and 3D silver nanovoid substrates on PDMS at 10 nm thickness indicates that the localized plasmon generated by the bowl shape, parameter (iv), would be responsible for only about 34% of total SERS activity reported for nanovoid substrate. Thus, the bumps and wrinkles formed during the sputtering on PDMS template (parameter (iii)), play a major role on SERS activity at this thickness for both 2D and 3D structures. However, this trend changes dramatically at 20 nm thickness, where the SERS intensity of 3D nanovoid substrate is 11.6 times higher than the 2D flat silver substrate on PDMS. Combined with the observation that there is no SERS activity reported at 20 nm thickness 2D flat silver substrate on glass slide, it is likely that there are only negligible thickness- and roughness-related plasmon localization effects (parameters (i), (ii), and (iii)), and the bowl shape-related localized plasmon resonance (parameter (iv)), starts to play a major role on the SERS activity for silver layers thicker than 20 nm. For silver film thicknesses larger than 30 nm, the SERS intensity does not change much and there are only negligible SERS intensities on both 2D flat silver substrates on PDMS and glass templates. This demonstrates that the SERS activity related to the roughness caused by the deformation of PDMS only takes place when the thickness of the silver film is less than 20 nm thick.

**Figure 5:**
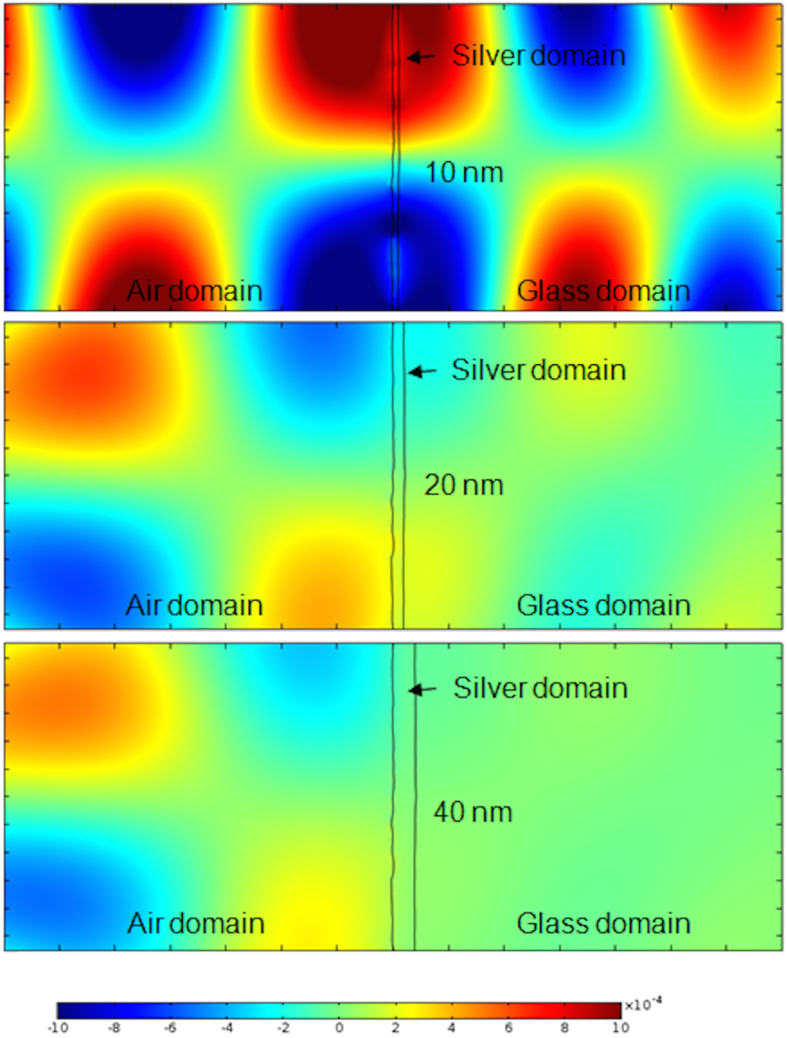
FEM simulations of the electric field (y-component) in air, silver, and glass domains (from left to right), with incident light coming from the left side (air), perpendicular to the air-silver interface. The air-silver interface profile used here is obtained from the AFM image of the silver surface (Fig. 2D), and the silver-glass interface profile is obtained from the AFM image of the glass surface (Fig. 2C). From top to bottom, these interfaces are attributed to silver layers of 10, 20, and 40 nm thickness.

To understand the effect of surface roughness and thickness of silver films on SERS activity, we preformed finite element method (FEM) simulations evaluating magnetic and electric fields of thin layers of silver deposited on glass. FEM simulations have been used in the past to verify and support the SERS data obtained experimentally for nanoparticles of different size and shape^35^,^36^,^37^, or to study the effect of the dielectric substrate on which they rest^38^. For this work, to ensure the simulation accuracy in modeling surface plasmon resonance, the results of Yushanov *et al*.^39^ were reproduced and then the parameters were modified according to our experiment. Two sets of simulations were performed. One set is using a perfectly flat surface without any roughness on the surface and the other set uses a real AFM-recorded surface with roughness values acquired from the AFM analysis of data presented in Fig. 2C,D. These flat and rough surface profiles were used to create the boundaries between the domains. The AFM silver surface profile of silver on glass was used for the air-silver interface and the AFM glass slide surface profile was used for the silver-glass interface. Silver domain thickness values of 10, 20 and 40 nm were simulated and presented in Fig. 5. The results of rough surface simulation on the electric field shown in Fig. 5 illustrate the relationship between local field strength and increasing thickness of deposited silver. It is obvious that the local field strength at the surface of the air-silver boundary increases with the thickness of the silver layer, which correlates well with the experimental results presented in Fig. 4C. The same trend was observed for the x-component of the electric field distribution and z-component of the magnetic field distribution (Figure S5). In addition, we present in Figure S6 the simulation of the electric field change for perfectly flat interfaces of different silver domain thicknesses. As seen in Figure S6, the thickness of deposited metal increases, the local field strength at the air-silver interface becomes stronger. These results suggest that the enhanced SERS activity observed experimentally for thinner films does not come from directly from the thickness of the metal and leads to the conclusion that the surface roughness for glass and silver film plays a significant role in the SERS activity.

FEM simulations were also used to explore the effect of excitation wavelength on the electric field enhancement. The simulations performed at 633 nm and 785 nm are presented in the supplementary materials and indicate that the 785 nm excitation yields a similar behavior as the 633 nm excitation, with highest enhancement at 10 nm silver film thickness (Figure S8).

These data suggests that the increased strength in the electric field (Fig. 5) and the increased SERS intensity (Fig. 4C) at smaller thicknesses for the rough silver film may result from larger values of the ratio between the roughness of the silver surface and the mean silver thickness. For the same roughness, as the mean silver thickness increases, the ratio between these two parameters decreases and becomes less relevant for SERS activity. Since PMDS exhibits even higher surface roughness, the ratio between the roughness and silver thickness is higher and therefore we expect, and observe here experimentally, higher SERS intensities for these substrates.

## Conclusions

In this paper we evaluated the effects of four physical parameters of 2D and 3D silver SERS substrates prepared by the sputtering process on the SERS activity. These parameters are: (i) sub-10 nm range clusters formed by sputtering, (ii) larger-sized wrinkles and bumps formed on PDMS templates during the sputtering process by shrinking and degassing of the soft PDMS substrate, (iii) thickness of the silver film, and (iv) nanovoid structures on PDMS template casted by latex beads. The 2D flat silver substrates on glass slides showed that sub-10 nm range nanoclusters have a negligible effect on SERS and only showed minor SERS activity at 10 nm thickness. The 2D flat silver substrate on PDMS template showed stronger SERS activity at thicknesses of 10 and 20 nm and only negligible activity at thicker silver coatings, suggesting that larger-sized nanostructures created on PDMS during the sputtering process contribute to the SERS activity only when accompanied by plasmon localization mediated by very thin films. 3D silver nanovoid substrates showed an order of magnitude stronger SERS activity at >20 nm silver nanofilms over 2D flat silver substrates on PDMS template, while showing only 34% higher SERS activity at 10 nm thickness, suggesting that additional plasmon localization occurs due to the nanovoid structures. Supporting FEM simulation results support the experimental trend of increasing field strength with decreased thickness of the deposited metal. These data demonstrate the interplay between surface roughness and thickness of the silver film on SERS activity and suggest that, for the same roughness, the thickness plays a significant role and should be considered as an important parameter in designing SERS substrates.

## Methods

### Materials used

DCM (dichloromethane) and ethanol were purchased from Sigma (St. Louis, MO), PDMS (polydimethlysiloxane) elastomer kit was purchased from Dow Corning (Carrollton, KY) and sulfate latex polystyrene beads were purchased from Invitrogen (Grand Island, NY).

### Fabrication of flat and nanovoid structure on PDMS template

We have followed the previously published protocol^26^. In short, to obtain PDMS nanovoid templates, 10 μL solution of 1 μm sulfate latex polystyrene beads was dropped on the clean glass slide surface and dried in the oven (100 °C) for approximately 10 minutes. The dried spot is roughly 3 ~ 4 mm in diameter. Pre-mixed PDMS elastomer and initiator (10 to 1 ratio) was then poured gently on the dried latex beads layer on top of the glass slide and cured for 2 hours in the oven at 100 °C. After 2 hours, the hardened PDMS layer was peeled off from the glass slide and washed with DCM to remove any latex beads bound to the PDMS surface. Highly uniform nanovoid structures of approximately 800 nm in diameter and 400 nm in depth were obtain in this way on the PDMS. The area outside the nanovoid region was flat PDMS.

### Silver nanofilm sputtering on various templates

Flat and nanovoid structured PDMS templates, as well as flat regular glass slides were sputtered with silver at different thicknesses of 10, 20, 30, 40 and 60 nm using a Kurt J. Lesker sputterer (Jefferson Hills, PA) at 10 mTorr and 300 mW.

### Surface characterization

AFM and SEM were used for the structural characterization of the fabricated structures. All SEM images were obtained using a Hitachi S-4100T (Tokyo, Japan) instrument, and AFM images were obtained using a MFP3D from Asylum Research (Goleta, CA).

### UV-Vis absorption spectroscopy

UV-Vis absorption spectra were measured by placing the nanovoid and flat area of the silver films perpendicular to the light beam in a Varian Cary 50 Bio UV-Vis spectroscopy from McKinley Scientific (Sparta, NJ).

### SERS measurements with 4-ATP as reporter molecule

4-ATP was prepared as 1 mM solution in ethanol. Then, SERS substrates (silver coated PDMS nanovoids, flat PDMS, and flat glass silde) were dipped in the 4-ATP solution for 1.5 hours. The substrate was then washed with ethanol twice to ensure that no residual 4-ATP is left on the substrate. SERS was measured using a Renishaw InVia Raman microscope (Gloucestershire, UK) with excitation wavelength at 633 nm and a 50X, 0.75NA objective lens that determines a spot size of approximately 1.25 micrometers. The exposure time was 10 s and laser power (measured after the objective) 42 μW for most substrates and 210 μW for glass template substrates. The measurements were performed in 10 different arbitrarily chosen spots (1 spectrum in each spot) of the substrate, and averaged for data presentation.

### Finite Element Method (FEM) Analysis

Simulations using COMSOL Multiphysics software (Palo Alto, CA) were performed to show the local field distributions on the surface of the deposited silver. A three domain system was constructed to simulate the air-silver-glass materials. A Transverse Magnetic (TM) wave, lambda = 633 nm, incident from the air domain serves as the excitation source. The angle of incidence of the TM wave to the silver domain is set to 90 degrees. The simulations solve Maxwell’s equations these domains with input values for the relative complex permittivity of air, silver and glass, respectively. The boundaries between the domains are simulated for different roughness effects and represent the air-silver and silver-glass interfaces of the substrates. The roughness of the different material interfaces is modeled by importing the data from the AFM scans. The AFM data is coordinate-transformed from z to x and x to y to fit the simulation geometry. The silver thickness parameter, d, is then varied and the local field distributions at the air-silver boundary were analyzed for thicknesses of 10, 20, and 40 nm. Analysis of the results was done by comparing the magnetic field z component and the electric field x and y components for different values.

## Additional Information

**How to cite this article**: Lee, C. *et al*. Thickness of a metallic film, in addition to its roughness, plays a significant role in SERS activity. *Sci. Rep*. **5**, 11644; doi: 10.1038/srep11644 (2015).

## Supplementary Material

Supplementary Information

## Figures and Tables

**Table 1:** Surface roughness (R_a_) of the silver film on flat PDMS and glass slide (nm).

Ag nano-film thickness	R_a_ of Ag film on flat PDMS	R_a_ of Ag film on glass slide
0	1.3a)	0.57b)
10	9.3	1.2
20	6.4	1.1
40	11.5	1.7

^a)^Flat PDMS template without the sputtering process;

^b)^Flat glass slide without the sputtering process.
