# Supplementary material for: Thickness of a metallic film, in addition to its roughness, plays a significant role in SERS activity
Source: Sci Rep. 2015 Jun 29;5:11644. doi: 10.1038/srep11644 (PMC5155581; doi:10.1038/srep11644)
Supplement: Supplementary Information [file srep11644-s1.pdf]

Supporting Information

Thickness of a metallic film, in addition to its roughness, plays a significant role in SERS activity

*Changwon Lee[1], Christopher S. Robertson[1], An H. Nguyen[1], Mehmet Kahraman[2], Sebastian Wachsmann-Hogiu[1,3]**

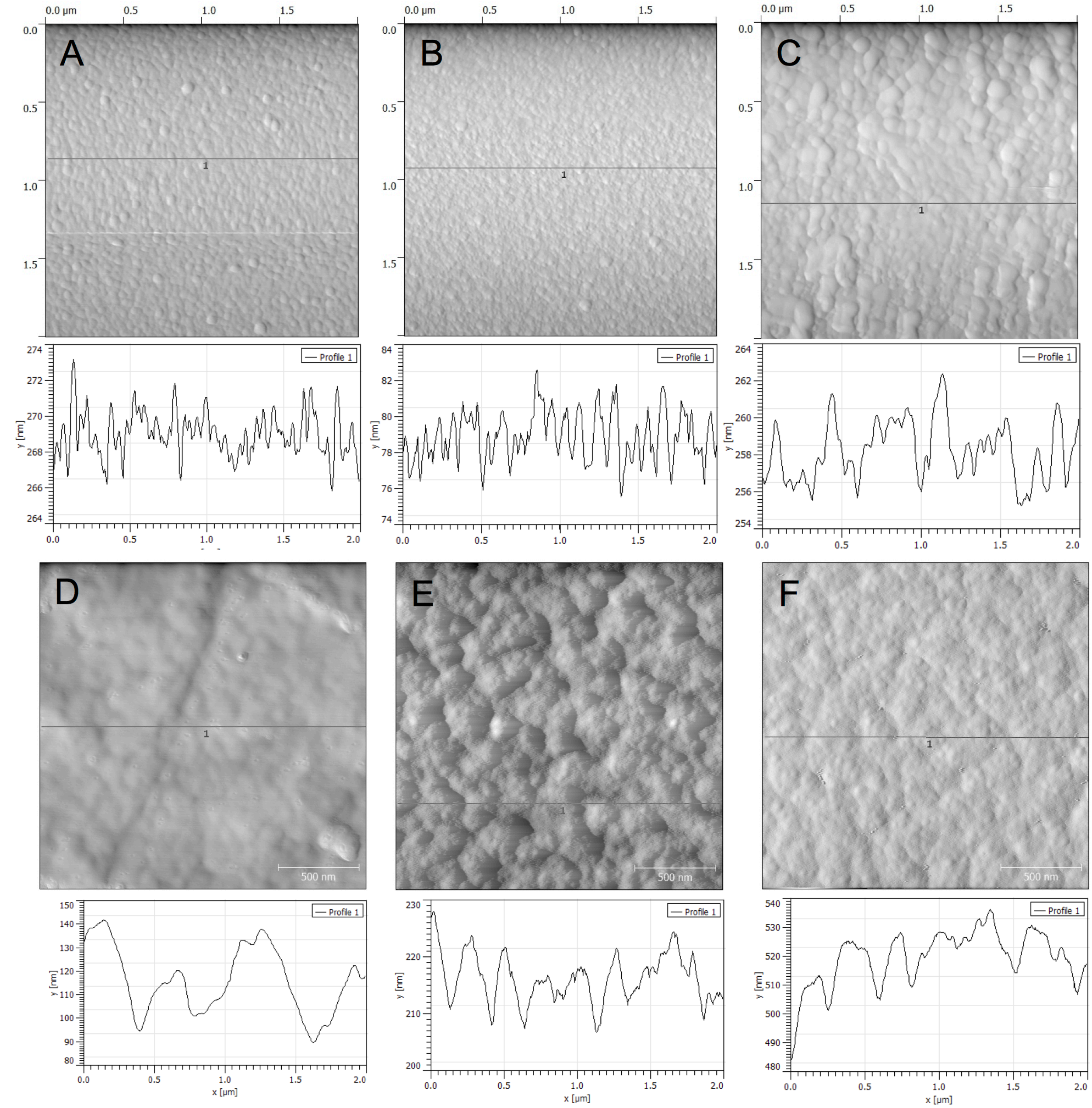

Figure S1. AFM images of sputtered silver on glass slides with different coating thicknesses of (A) 10 nm, (B) 20 nm, and (C) 40 nm and on flat PDMS templates of (D) 10 nm, (E) 20 nm, and (F) 40 nm.

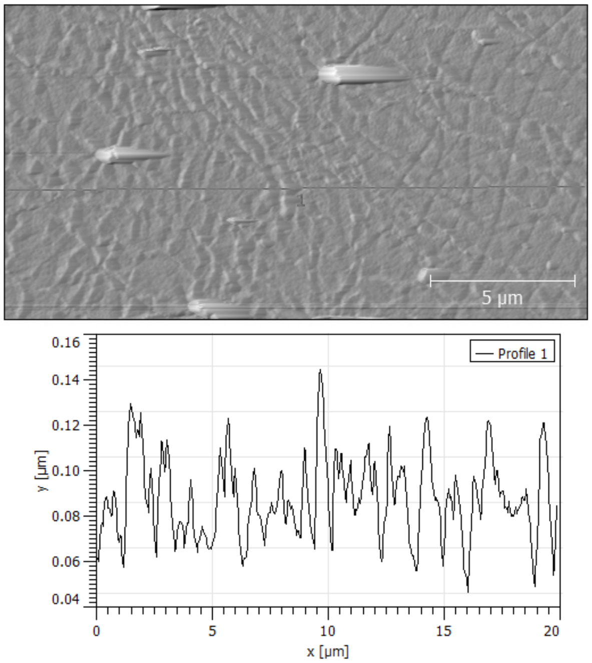

Figure S2. Wider range view of a 10 nm thick 2D silver substrate on PDMS template (20 µm x 10 µm). Large-scale wrinkles and bumps are visible throughout the image.

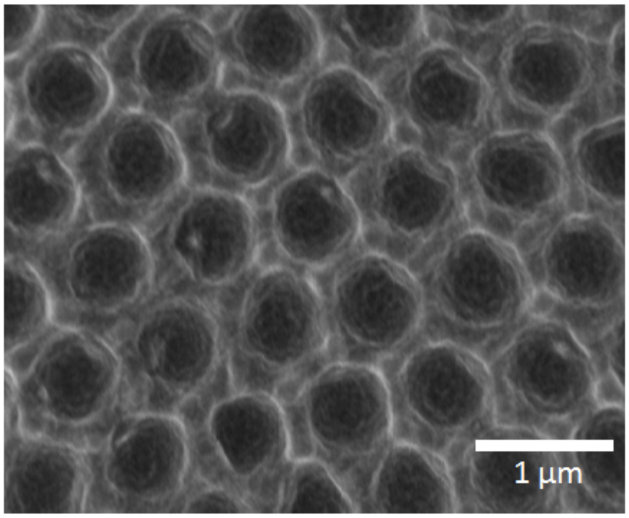

Figure S3. SEM image of a 10 nm silver sputtered nanovoid structure.

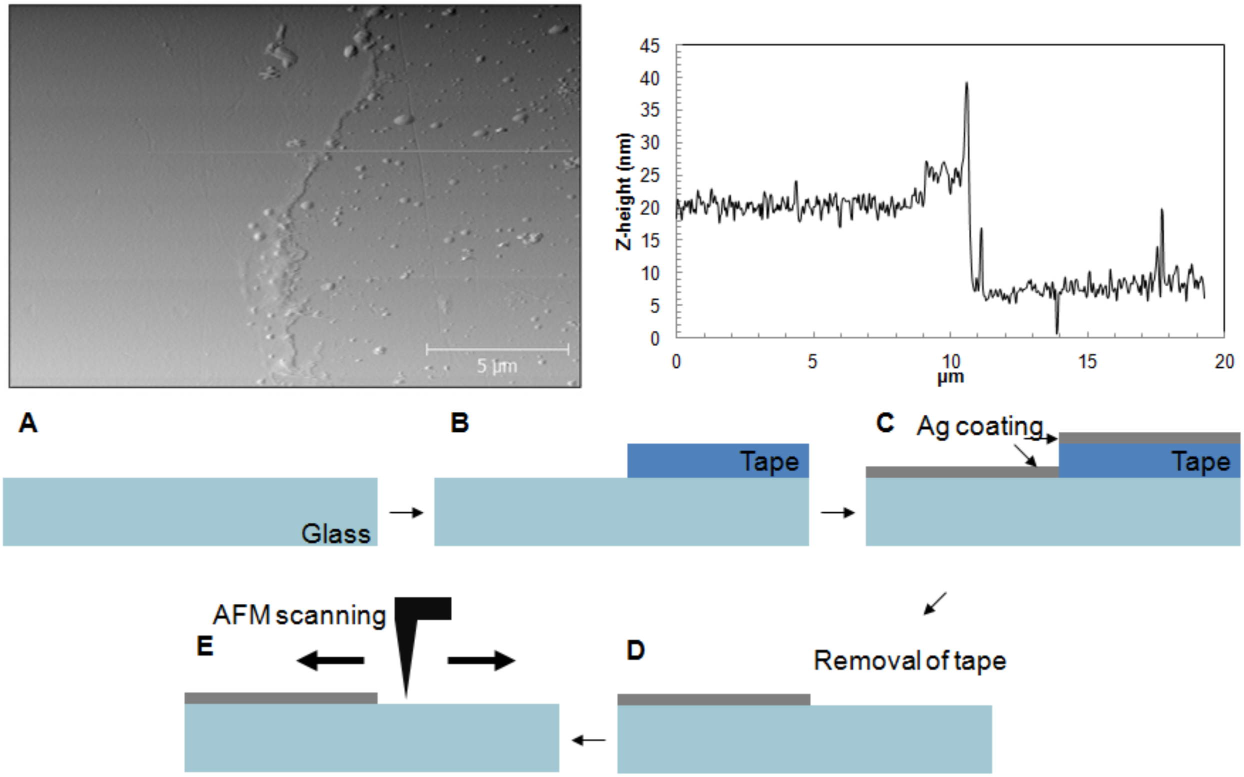

Figure S4. AFM image (top left) showing the continuity profile (top right) of the 10 nm silver coating on a glass slide. Left side of the AFM image shows the silver nanofilm layer and right side shows bare glass surface with scotch tape adhesive residues shown as bumps. The edge of the silver nanofilm (shown as increased height in the z-profile) is curled up as the tape was removed upward. The schematic shown in A-E illustrates the process of sample preparation and AFM measurement that demonstrates the uniformity of the silver layer.

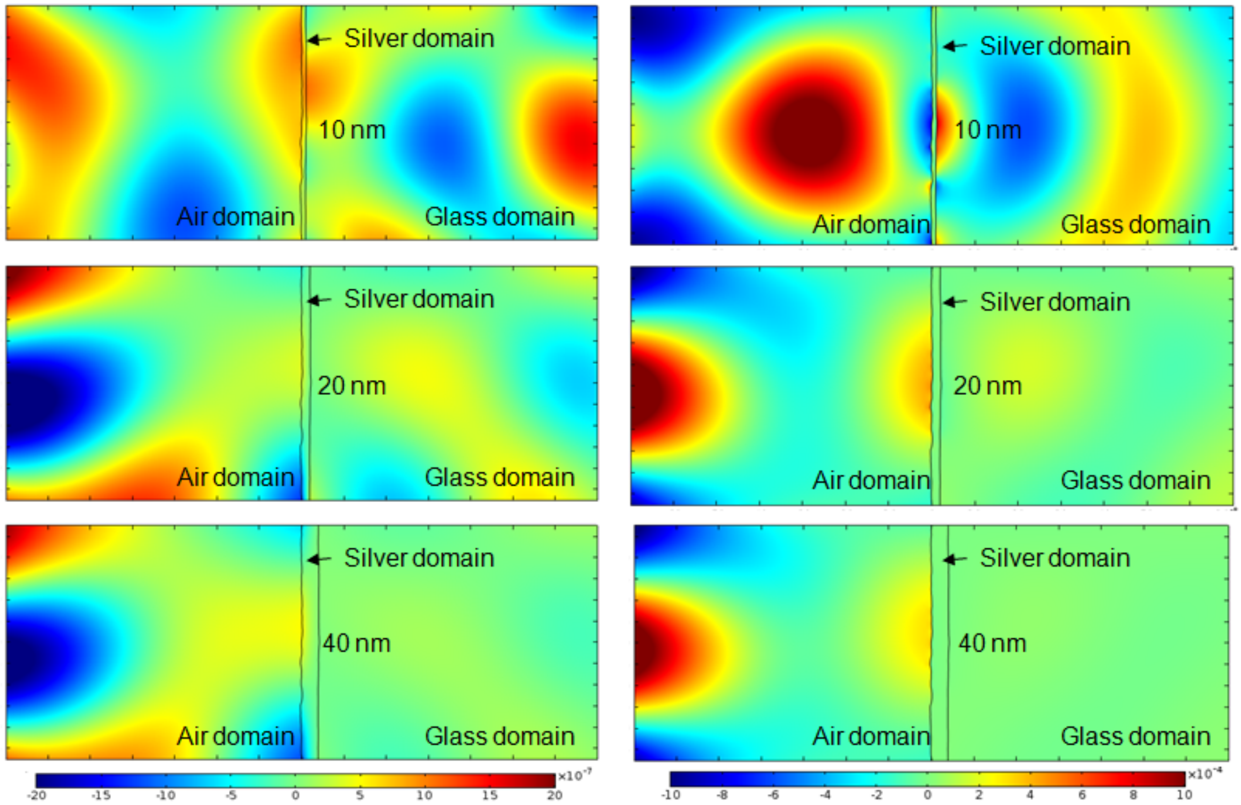

Figure S5. FEM simulations of the magnetic field (z-component, left side column) and electric filed (x-component, right side column) of rough interfaces with silver thicknesses of 10, 20, and 40 nm. The interface profiles are obtained from the AFM analysis of the silver on glass and glass surfaces.

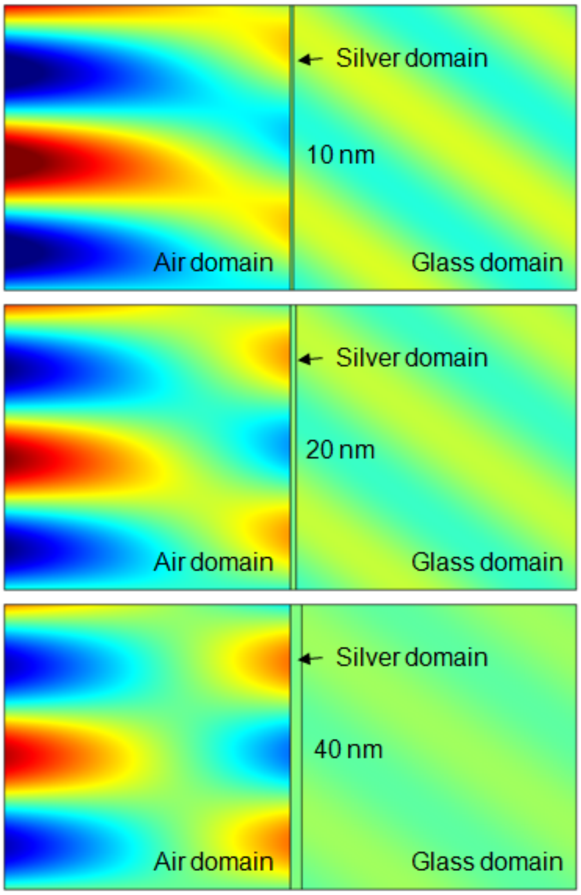

Figure S6. FEM simulations of the air-silver-glass domains (from left to right) of perfectly flat interfaces with different silver domain thicknesses of 10, 20, and 40 nm.

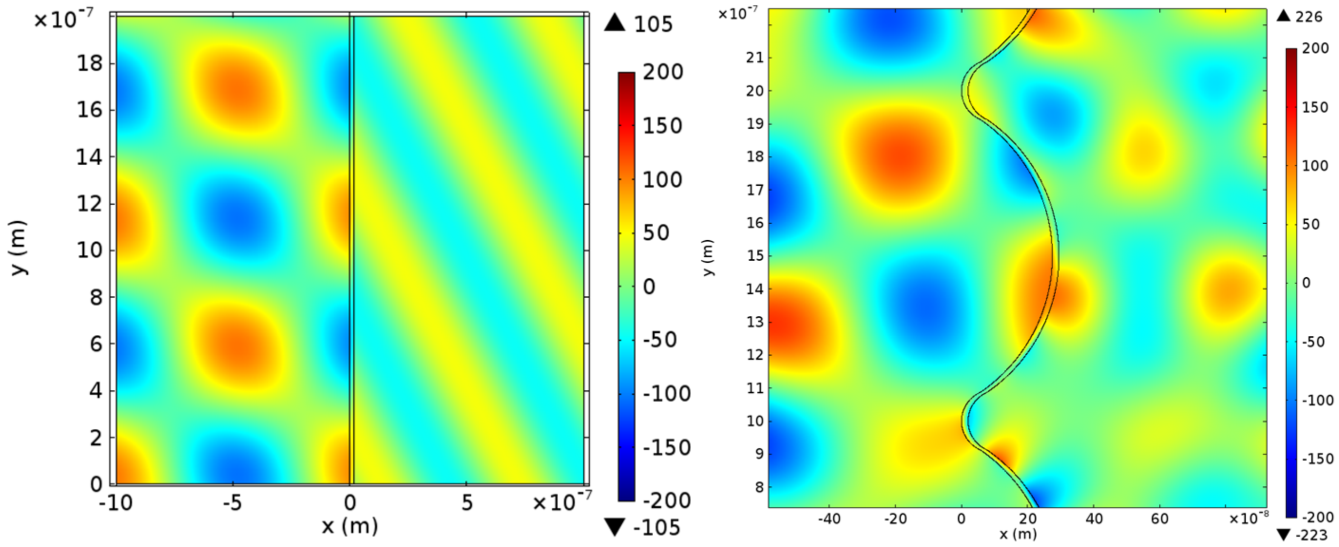

Figure S7. Simulations of smooth flat and nanovoid profile of air (left)-silver-PDMS (right) layers. Note that the maximum color scale for flat profile is 105 and that of nanovoid profile is 226, indicating higher enhancement for the nanovoid structure, due to far-field contributions.

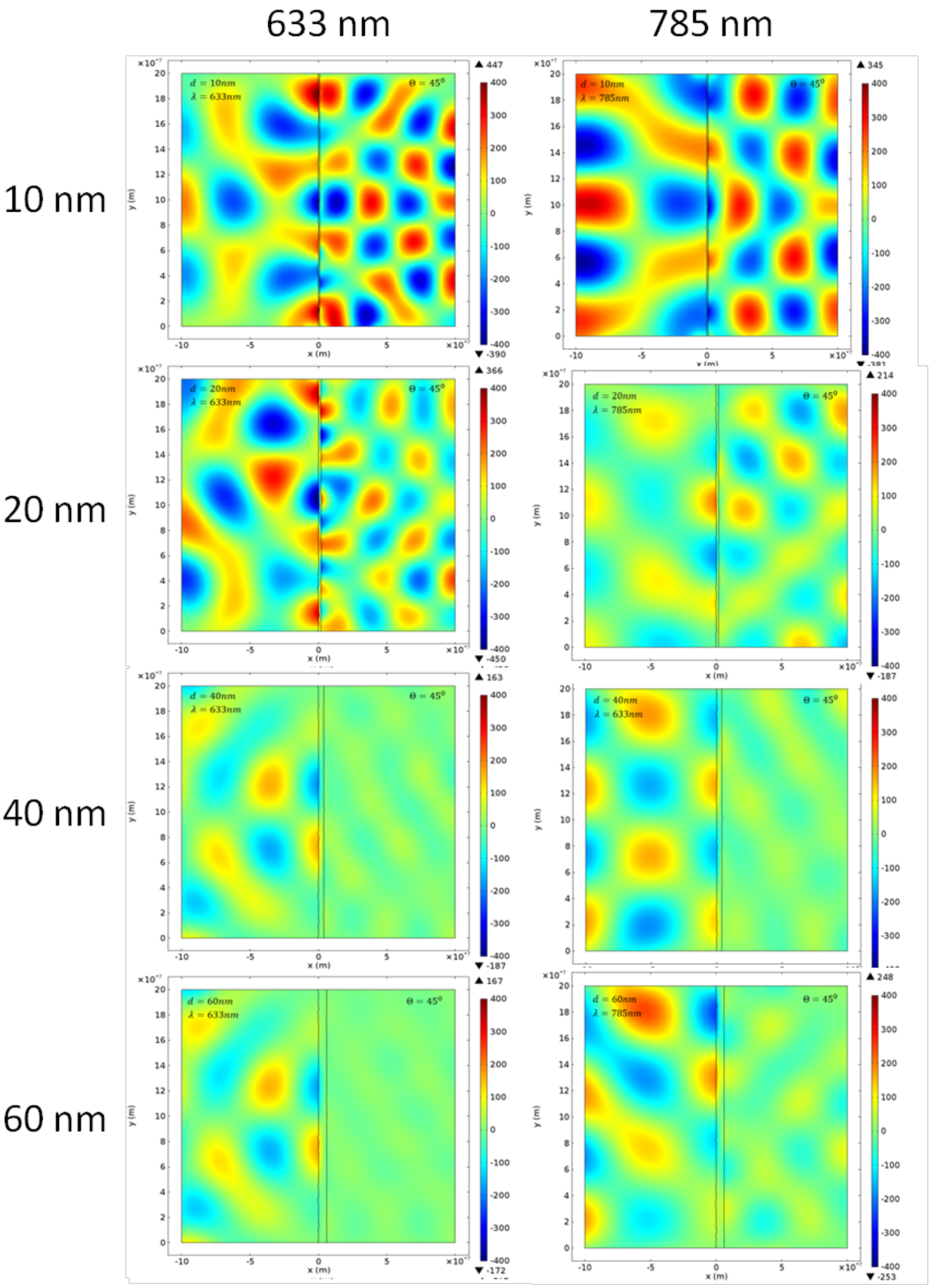

Figure S8. Simulation of Ag on glass at different silver thickness of 10, 20, 40, and 60 nm with two different excitation wavelengths of 633 and 785 nm. The stripe in the middle represents the silver layer that has air to the left and glass to the right.
